# Supplementary material for: E-Cadherin/HMR-1 Membrane Enrichment Is Polarized by WAVE-Dependent Branched Actin
Source: J Dev Biol. 2021 May 7;9(2):19. doi: 10.3390/jdb9020019 (PMC8162361; doi:10.3390/jdb9020019)

Table S1. Embryonic lethality caused by the RNAi

Genotype	# Trials	Alive	Dead	Total	% Dead
hmr-1 RNAi	3	338	305	643	47
gex-3 RNAi	5	65	1296	1361	95
hmp-2 RNAi	3	430	278	708	39
hmp-1 RNAi	1	361	27	388	7
dlg-1 RNAi	1	211	119	360	36
gex-2 RNAi	1	182	283	465	61

Table S2. GFP knockdown by the RNAi

Genotype	N	Mean	% Decrease	Max	% Decrease
hmr-1::gfp	6	546		1925	
hmr-1::;gfp; hmr-1 RNAi	6	107	80	291	85
unc-40 gex-3; gex-3::gfp	9	218		449	
unc-40 gex-3; gex-3::gfp; gex-3 RNAi	7	55	75	127	72
hmp-1::gfp	6	420		911	
hmp-1::gfp; hmp-1 RNAi	6	367	13	864	5
hmp-2::gfp	10	391		972	
hmp-2::gfp; hmp-2	13	380	3	742	24

Figure S1. GFP Knockdown with RNAi

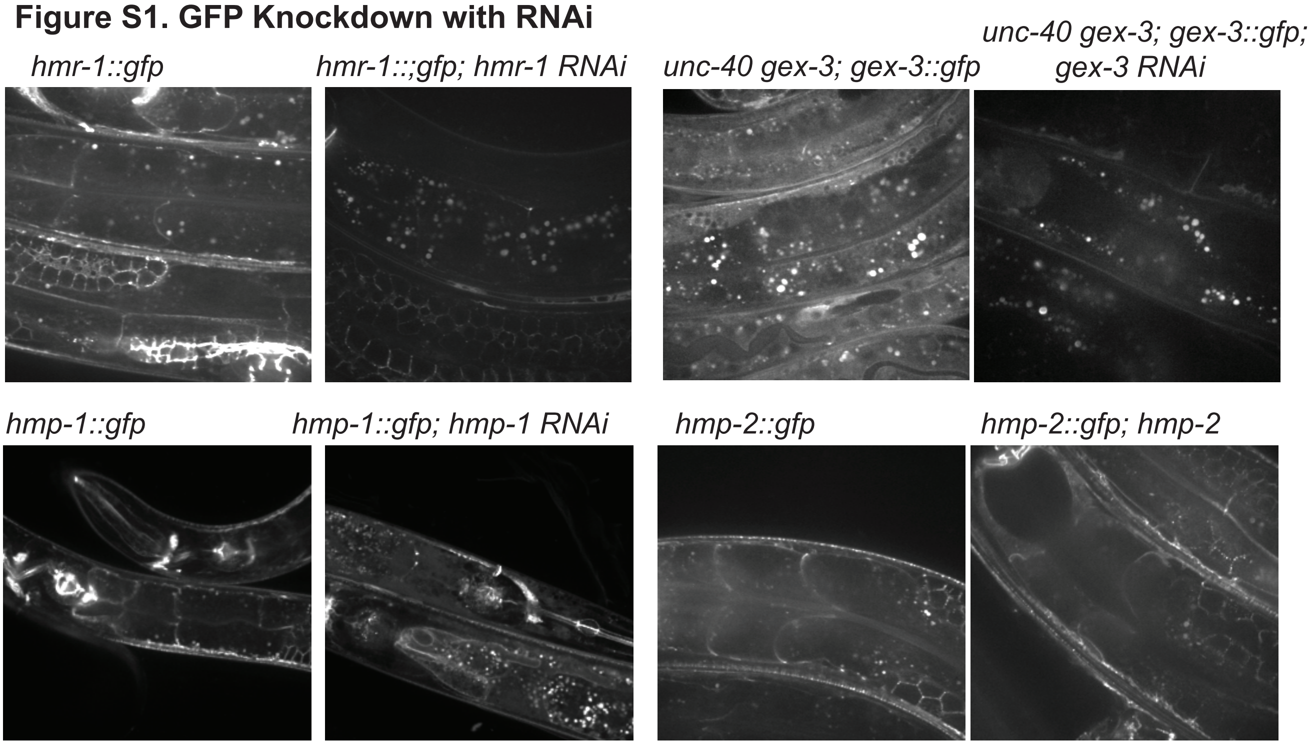

Supplement: Supplementary file 1 [file jdb-09-00019-s001.zip › jdb-1179780-SI.pdf]
